# Local Corrosion Behaviors in the Coarse-Grained Heat-Affected Zone in a Newly Developed Zr–Ti–Al–RE Deoxidized High-Strength Low-Alloy Steel

**DOI:** 10.3390/ma16020876

**Published:** 2023-01-16

**Authors:** Chao-Chao Yin, Lin Cheng, Zhi-Hui Wang, Tian-Liang Zhao, Shi Cheng, Shu-E Hu, Zi-Cheng Liu, Deng Luo, Da-Heng Xiao, Xing Jin, Han-Kun Liu, Kai-Ming Wu

**Affiliations:** 1 The State Key Laboratory of Refractories and Metallurgy, Hubei Province Key Laboratory of Systems Science on Metallurgical Processing, International Research Institute for Steel Technology, Collaborative Center on Advanced Steels, Wuhan University of Science and Technology, Wuhan 430081, China; 2 Iron and Steel Institute, Shandong Iron & Steel Group Rizhao Co., Ltd., Rizhao 276800, China; 3 Department of Manufacturing, Baoshan Iron & Steel Co., Ltd., Shanghai 201999, China; 4 Technology Center, Hunan Valin Xiangtan Steel Co., Ltd., Xiangtan 411101, China; 5 Department of Manufacturing, Nanjing Iron & Steel Co., Ltd., Nanjing 210044, China; 6 Iron and Steel Institute, China Petroleum Group Ocean Engineering (Qingdao) Co., Ltd., Qingdao 266520, China

**Keywords:** inclusion, pitting corrosion, HSLA steel, CGHAZ, first-principles, rare earth elements

## Abstract

Oxide metallurgy technology can improve the microstructure of a coarse-grained heat-affected zone (CGHAZ) but introduces extra inclusions. Local corrosion behavior of the CGHAZ of a Zr–Ti–Al–RE deoxidized steel was investigated in this work using theoretical calculations and experimental verification. The modified inclusions have a (Zr–Mg–Al–Ca–RE)O_x_ core claded by a CaS and TiN shell. CaS dissolves first, followed by the oxide core, leaving TiN parts. This confirms that the addition of rare earth can reduce lattice distortion and prevent a galvanic couple between the inclusions and the matrix, while the chemical dissolution of CaS causes localized acidification, resulting in the pitting corrosion initiation.

## 1. Introduction

High-strength low-alloy (HSLA) steels are important in the construction of offshore platforms due to their weldability and high cracking resistance [1,2]. An important method for bonding steel for large structures in industrial applications is welding. High heat input is typically utilized to improve welding efficiency and lower production cost [3]. During the welding thermal cycle, the coarse-grained heat-affected zones (CGHAZs) experience a fast heating rate and a high heating peak temperature, resulting in the abnormal growth of the grains and the development of complex microstructures such as granular bainite (GB), lath bainite (LB), acicular ferrite (AF) and martensite–austenite (M/A) constituents. HSLA steels can be classified and defined according to their morphologies and characteristics. GB consists of elongated ferrite sheaves with blocky M/A constituents. LB is formed with parallel laths at fast cooling rates. AF is staggered, interlocked and formed on non-metallic inclusions. The M/A constituent is a mixture of untempered martensite embedded in carbon-enriched retained austenite [4,5]. As a result, the CGHAZ is considered to be the weakest area in the welded joint [6]. Oxide metallurgy technology is one of the effective solutions for introducing specific fine, non-metallic inclusion particles into steels to refine the grain size and/or induce the formation of a beneficial microstructure. The introduction of fine and uniform, non-metallic inclusion can inhibit the abnormal growth of grains in the heating process and induce the nucleation of acicular ferrite in the cooling process [2,7]. Thus, the application of oxide metallurgy technology is promising for refining the microstructure and improving the mechanical properties of the CGHAZ.

Generally, the types of non-metallic inclusions—such as Al_2_O_3_, MnS, ZrO_2_, CaO and Al_2_MgO_4_—introduced in steels depend on the specific deoxidation techniques [8,9,10,11,12,13,14,15]. Inclusions in the steels are often considered as defects that can preferably be attacked by chloride ions in the marine environment, which induce pitting corrosion [4,16,17] that has a detrimental impact on application performance [18,19,20,21,22,23]. Numerous studies have been conducted on the local corrosion caused by inclusions in HSLA steels. Some researchers believe that galvanic corrosion could occur between the inclusions and the adjacent matrix. Hou et al. investigated the pitting behavior of Al–Ti–Mg deoxidized steel and found that MnS had a lower surface potential than Al_2_O_3_ and Al_2_MgO_4_ and acted as an anode more easily [8]. Wei et al. found that pitting corrosion behavior is related to the microgalvanic effect between MnS and the matrix [15]. However, Liu et al. found that most of the non-metallic inclusions have weak conductivity. Their research shows that ZrO_2-_Al_2_O_3_-Ti_2_O_3_ inclusions do not form a galvanic couple with the adjacent matrix [24]. Instead, the main factors that are assumed to induce pitting behavior are the microcracks and high dislocation density areas around the irregular Al_2_O_3_. In addition to the shape, type and conductivity of the inclusions, the distribution and size of the inclusions have a significant impact on the pitting initiation. Normally, the finer the inclusions, the higher the pitting potential and the greater the contribution to the pitting resistance of the steel [18].

Rare earth treatment is often used by researchers to effectively improve the corrosion resistance of steels because the rare earth (RE) elements have a strong affinity to oxygen and sulfur, capable of refining and spheroidizing the inclusions and densifying the corrosion layer [18,25,26,27,28]. In a simulated marine environment, Liu et al. analyzed the pitting behavior of a Q460NH steel treated with RE and found that the pitting corrosion starts from the (RE)_2_O_2_S-(RE)_x_S_y_ rather than the matrix, suggesting that the matrix after RE treatment has higher corrosion resistance than the inclusions in the simulated solution [29]. Liu et al. discovered that the initiation of pitting corrosion in Zr–Ti deoxidized low-alloy steel was caused by matrix dissolution caused, in turn, by lattice distortion and microcrevices around ZrO_2_-Ti_2_O_3_-Al_2_O_3_ inclusions. After adding rare earth, the lattice distortion of inclusions decreased dramatically and the dissolution of the (RE)_2_O_2_S-(RE)_x_S_y_ caused pitting corrosion [30]. Using the first-principles theory, Tang et al. calculated the work function of rare earth oxysulfide and confirmed that rare earth sulfide is easier to dissolve, from an atomic scale perspective, than rare earth oxide [31]. The mechanism of inclusion-induced local corrosion is still controversial. Whether the composite inclusions and the matrix constitute galvanic corrosion, crevice corrosion or the dissolution order needs to be further investigated [8,10,15,24,29,32,33,34,35].

Previous studies demonstrated that the CGHAZ exhibits a higher corrosion tendency than the base metal and weld metal and is more likely to trigger anodic dissolution (AD) behavior in the entire welded joint because of its coarse grains, complex microstructure [36,37,38], welding residual stress and crystal defects [16,39,40]. The application of oxide metallurgy technology may change the environment where corrosion occurs in the CGHAZ. For example, use of oxide metallurgy technology, such as Widmanstätten ferrite in the CGHAZ, will affect the microstructure formed in the welding thermal cycle and can improve corrosion resistance [38]. It also introduces different types of inclusions, and the difference in electrochemical information on the surface of these inclusions may affect pitting initiation behavior. In addition, oxide metallurgy techniques may lead to the distribution of aggregated inclusions in the matrix, which may cause an effective increase in the active sites of pitting initiation. Thus, to improve the mechanical properties of the CGHAZ, oxide metallurgy technology and rare earth microalloying often result in more complex inclusions, posing challenges in the study of the corrosive behavior of inclusions [11,13,41,42].

In this paper, the achievement of oxide metallurgy technology in modifying and refining inclusions and improving the microstructure of the CGHAZ in an HSLA steel were first evaluated. Next, the formation and evolution of inclusions in CGHAZ during the welding process were investigated, followed by the study of the pitting behavior of Zr–Ti–Al–RE composite inclusions in a CGHAZ, in a simulated marine environment, by means of an immersion test, use of a scanning electron microscope equipped with an energy dispersive spectroscope (SEM-EDS) and density functional theory.

## 2. Experimental Section

### 2.1. Sample Preparation and CGHAZ Simulation

Table 1 displays the chemical composition of the 12 mm-thick tested steel, which was deoxidized by Zr–Ti–Al–RE, where the RE elements refer to La and Ce. The steel was produced by thermomechanical controlled processing (TMCP) in a domestic steel factory in China. The microstructure in CGHAZ was generated using the Gleeble 3800 Thermo-Mechanical simulator (Dynamic Systems Inc., Poestenkill, NY, USA) with a heat input of 100 kJ/cm for submerged-arc welding. Machined samples with dimensions of 11 mm × 11 mm × 75 mm were reheated to peak temperature of 1320 °C at a rate of 200 °C/s, maintained for 2 s and then cooled according to Rykalin 3D models (formulation (1)) [43]:

(1) $$t_{8/5}=\frac{E}{2\pi \lambda }\left[\left(\frac{1}{500-T_{0}}\right)-\left(\frac{1}{800-T_{0}}\right)\right]$$

where *E* is heat input, kJ/cm, *λ* is thermal conductivity, J/(cm·s·°C), and *T*_0_ is the initial temperature, °C. The *t*_8/5_ corresponding to 100 kJ/cm heat input was 45 s.

### 2.2. Characterization of Microstructure and Complex Inclusions

To analyze the microstructure in CGHAZ, samples with dimensions of 10 mm × 10 mm × 5 mm, which were cut from the welding thermal cycle test, were ground with SiC paper from 200 to 2000 grits. The samples were then polished with diamond paste (2.5 μm), cleaned quickly with ethanol and, finally, etched with 4% nital (4 mL HNO_3_ + 96 mL C_2_H_5_OH). The morphology was observed using a scanning electron microscope (SEM, FEI Nova400, Lincoln, NE, USA) and transmission electron microscope (TEM, JEOL JEM-2100F, Akishima, Japan). Several inclusions were separated from the matrix by electrolysis in non-aqueous solution (pH ≈ 8) at 0~5 °C for further chemical composition and morphology observation [44]. 500 mL of the electrolyte solution was prepared, which consisted of 5.0 g of tetramethylammonium chloride, 3.0 g of anhydrous barium oxide, 50 mL of acetylacetone and 450 mL of anhydrous methanol. A scanning electron microscope (SEM, FEI Nova400) with energy dispersive spectroscopy (EDS) was used to scan random positions on the polished sample surface to characterize the chemical composition, morphology and particle size of inclusions. For the subsequent electron backscattered diffraction (EBSD) tests, the test samples were polished with diamond paste (2.5 µm) and then ionized by milling using GATAN 685.O (Pleasanton, CA, USA). The EBSD test was performed using a field emission scanning electron microscope (FE-SEM) at a voltage of 20 kV, a current of 13 nA and a step size of 0.1 µm. The data was analyzed by OIM software (Version: 5.3) to obtain the inverse pole figure (IPF) map, kernel average misorientation (KAM), image quality (IQ) diagrams to determine the crystal orientation of microstructure, and lattice distortion between the inclusions and matrix. The JMatPro software (Version: 7.0) was utilized to calculate the precipitation behavior of inclusions during the solidification process (1600–600 °C). Origin 8.0 software was used to plot the solid–solution behavior of the nanoparticles and temperature during the welding thermal cycle.

### 2.3. Electrochemical Tests

The potentiodynamic polarization tests, which employed a three-electrode cell composed of working electrode, counter electrode (platinum plate electrode) and reference electrode (saturated calomel electrode (SCE)) at room temperature, were conducted on electrochemical workstation (E4, Kronach Zahner, Germany). The 500 mL solution contained 3.5 wt.% NaCl (Chinese Medicine Chemical Reagent Co., Ltd., Shanghai, China). The potentiodynamic scans began at −300 mV vs. OCP and continued until the current density reached 1 mA·cm^−2^. The scan rate was 0.333 mVs^−1^. All electrochemical measurements were performed more than three times for greater reproducibility [31,45]. Electrochemical impedance spectroscopy (EIS) tests were performed at a frequency ranging from 10^5^ Hz to 0.01 Hz with amplitude of 10 mV. ZView software (Version: 3.30d) was used to analyze impedance data of Nyquist and Bode plots.

### 2.4. Immersion Test

To investigate the effect of Cl^−^ on the pitting initiation and propagation of composite inclusions by ex situ SEM observations in a simulated marine environment, a series of immersion tests (5 s, 1 min, 1 h and 2 h) were conducted. To better observe the pitting behavior of inclusions, the immersion test was conducted in 0.1 wt.% NaCl and the rust on the surface of the immersed sample was removed by using a solution of 50% HCl + 50% deionized water + 5 g/L C_6_H_12_N_4_ [46]. An FE-SEM with EDS was used to analyze the local corrosion morphology of the specimens before and after rust removal. For each stage of localized corrosion, the observation experiment was repeated at least three times, and at least five areas were observed in each sample.

### 2.5. First-Principles Calculation

The physical properties of the inclusions and body-centered cubic Fe (BCC), such as density of states, elastic constant and work function, were calculated to better elucidate the local corrosion mechanism. The conductivity of inclusions is explained by the density of states calculation. Furthermore, this investigates whether there is a microgalvanic effect between the inclusions and the matrix. The elastic constants were calculated to determine if the differences in physical properties between the inclusions and the Fe matrix might cause stress concentrations or the development of microcracks. The work function (WF), which represents the ability of electrons to escape to the metal surface, is used to assess the corrosion resistance of inclusions in tested steel. All calculations are based on density functional theory (DFT) and are performed with the Vienna ab initio simulation package (VASP) and the projector-augmented wave (PAW) method [47]. Here, the generalized gradient approximation (GGA) is adopted, and the Monkhorst–Pack grids are used in the Brillouin zone integration. All DFT models are guaranteed to have the same stoichiometric ratio as the molecular formula ratio. The setting of the K point roughly satisfies K * a (lattice constant) ≈ 45. The thickness of the vacuum layer is set to 15 Å. Dipole correction is applied along the thickest side. Depending on the type of inclusion, a cut-off energy range from 400 eV to 600 eV is selected. The convergence criteria for energy and force are set to 1.0 × 10^−5^ eV/atom and 0.01 eV/Å, respectively. The calculation of the density of states is conducted on the relaxed unit cell. The work function is expressed as follows (Equation (2)):

(2) $$WF=V_{e}-E_{f}$$

where *V_e_* represents the vacuum level and *E_f_* represents the Fermi level. Considering the relatively lower surface energy and more stable crystals of the low-index crystalline planes, three low-index crystalline planes, (100), (110) and (111), were selected to calculate the inclusions’ work function with the periodic slab model [31]. To simulate the bulk phase, a few atom layers at the bottom of the slab were fixed in their optimized positions. Meanwhile, at least two atomic layers were relaxed. As shown in Equations (3)–(10), the Voigt–Reuss–Hill (VRH) approximation method was used to determine the elastic mechanical properties of the inclusions and the matrix [48,49]:

(3) $$B_{V}=\frac{\left(C_{11}+C_{22}+C_{33}\right)+2\left(C_{12}+C_{13}+C_{23}\right)}{9}$$

(4) $$B_{R}=\frac{1}{\left(S_{11}+S_{22}+S_{33}\right)+2\left(S_{12}+S_{13}+S_{23}\right)}$$

(5) $$G_{V}=\frac{C_{11}-C_{22}+3C_{44}}{5}$$

(6) $$G_{R}=\frac{5\left(C_{11}-C_{12}\right)C_{44}}{3\left(C_{11}-C_{12}\right)+C_{44}}$$

(7) $$G=\frac{G_{V}+G_{R}}{2}$$

(8) $$B=\frac{B_{V}+B_{R}}{2}$$

(9) $$E=\frac{9BG}{3B+G}$$

(10) $$\mu =\frac{3B-2G}{2\left(3B+G\right)+G}$$

where *C_ij_* represents the elastic stiffness constant and *S_ij_* represents the elastic compliance constant. *B*_V_ and *B*_R_, and *G*_V_ and *G*_R_ are the bulk modulus and shear modulus, calculated by the Voigt and Reuss approximation methods, respectively. *G*, *B*, *E* and *μ* represent shear modulus, bulk modulus, Young ‘s modulus and Poisson ‘s ratio, respectively.

## 3. Results and Discussion

### 3.1. Formation and Evolution of the Composite Inclusions in CGHAZ

#### 3.1.1. Microstructure and Inclusion Analysis

**Figure 1:**
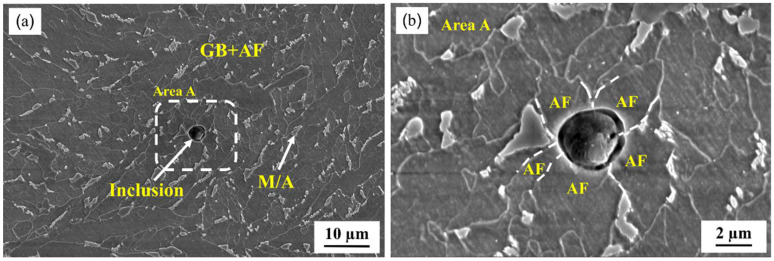
(**a**) Scanning electron microscopy microstructure of the CGHAZ., (**b**) morphology of the enlarged part in the white box in (**a**). (GB: granular bainite, AF: acicular ferrite, M/A: martensite–austenite constituents).

The microstructure was composed of GB and AF with blocky M/A constituents which were distributed discontinuously at the sub-boundary after etching with 4% nital for 8~12 s (Figure 1). At a heat input of 100 kJ/cm, the average impact energy at −20 °C of the simulated specimen reached 370 J, which indicates that Zr–Ti–Al–RE deoxidization guarantees the improved mechanical properties in the CGHAZ. Meanwhile, it was found that some inclusions prompted the formation of acicular ferrite. As shown in Figure 1b, several acicular ferrite grains emanated from a spherical inclusion, which could prevent or deviate the crack prorogation and improve the toughness [21,50]. High-strength low-alloy (HSLA) steels experience severe grain coarsening and loss of toughness as a consequence of welding heat input, particularly high heat input [5,51]. However, inclusions and nanoparticles such as TiN and Ti_2_O_3_ can effectively inhibit the austenite grain growth in the range of 1200–1350 °C and, during cooling, prompt the formation of ideal acicular ferrite, which could significantly improve the toughness of the CGHAZ [52,53].

**Figure 2:**
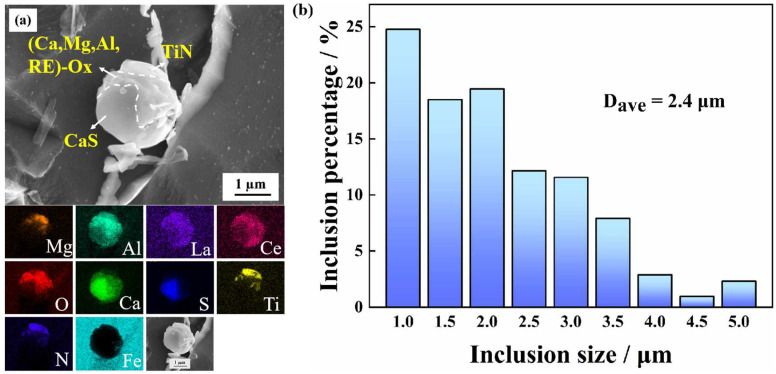
(**a**) SEM-EDS mapping images and (**b**) equivalent size distribution of inclusions.

To better observe and analyze the composite inclusions, they were separated by electrolysis in a non-aqueous solution (Figure 2a). In this work, the inclusions are found to be almost spherical in shape, forming a typical core–shell structure, with (RE–Mg–Al–Ca)–O_x_S_y_ in the center and CaS and TiN at the periphery. The addition of Zr, Ca and RE has an obvious spheroidization effect on the inclusions [54,55,56]. The appearance of Mg is attributed to the refractory bricks used in steelmaking. However, Zr was not detected on this exposed surface. Rare earth elements could induce various types of inclusions such as (Al, Zr, RE)-O_x_, (RE)_2_O_x_S_y_, and (RE)_x_S_y_ [31,55]. According to statistical analysis, (RE–Zr–Mg–Al–Ca)–O_x_S_y_–TiN composite inclusions are formed in this tested steel. Figure 2b shows the equivalent particle size distribution of the inclusions. Nearly 85% of the inclusions are less than 3 µm in diameter, with a total average diameter of the inclusions of 2.4 µm and a particle number density of 18.27 mm^−2^. It should be noted that the inclusions with a particle size less than 1 µm are not included in Figure 2b.

#### 3.1.2. Thermodynamic Analysis of Inclusions and Nanoparticles’ Evolution Behavior in CGHAZ

**Figure 3:**
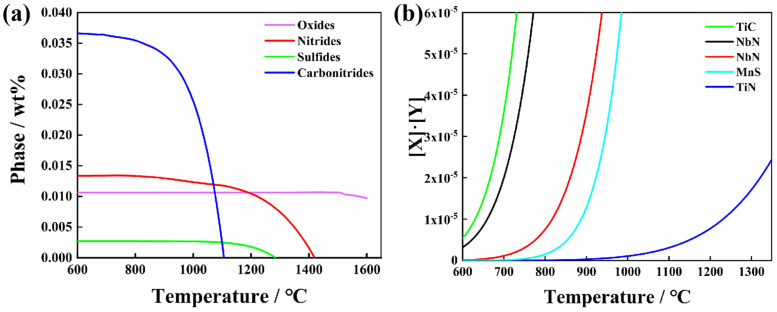
(**a**) Precipitation curves of the sulfide and nitride during the solidification process and (**b**) nanoparticle precipitation in welding thermal cycle.

Inclusions such as oxides, sulfides and nitrides can be formed during solidification, given the diversity of alloying elements in the tested steel. The change in Gibbs free energy of the formation reaction of the inclusions is the criterion for determining whether the reaction can proceed spontaneously at constant temperature and pressure; *ΔG* < 0 indicates that the reaction occurs spontaneously [25]. The chemical reaction equation and thermodynamic formula of the deoxidization reaction in molten steel are shown in Equations (11) and (12) [57]:

(11) $$\frac{x}{y}\left[M\right]+\left[O\right]=\frac{1}{y}\left(M_{x}O_{y}\right)$$

(12) $$\Delta G=\Delta G^{\theta }+RTlnK=\Delta G^{\theta }+RTln\frac{a_{M_{x}O_{y}}^{1/y}}{a_{\left[O\right]}a_{\left[M\right]}^{x/y}}$$

where *ΔG* and *ΔG^θ^* represent the Gibbs free energy and the standard Gibbs free energy, respectively, of the reaction, (J·mol^−1^), *R* is the gas constant, (J·mol^−1^·K^−1^), *T* represents the temperature, (K), and *a_i_* represents the activity of the element. Table 2 presents the corresponding free energy data for various formation reactions of the inclusions that may be involved in this work. According to Gibbs free energy change calculated at 1873 K, which is shown in Table 2, the single-metal oxide formation occurs in the following order: Al_2_O_3_ (−734.33 kJ/mol), La_2_O_3_ (−610.85 kJ/mol), Ti_2_O_3_ (−273.72 kJ/mol), ZrO_2_ (−156.09 kJ/mol) and CaO (−96.36 kJ/mol). Furthermore, Zr and RE can entirely or partially displace Al in Al_2_O_3_ in molten steel to form (ZrO_2_(−7298.12 kJ/mol) and LaAlO_3_(−646.15 kJ/mol)). Ca can react with solute aluminum and oxygen to form calcium aluminate (−9458.40 kJ/mol). Generally, Al_2_O_3_ dominates the initial inclusions for normal aluminum deoxidation technology used in steel making. Meanwhile, refractory bricks react easily with Al_2_O_3_ to generate Al_2_O_3_·MgO (similar to spinel) [58]. The inclusions are modified into (RE–Zr–Ti–Ca–Mg)–O_x_ with Zr, Ca, Ti and RE acting as deoxidizers [59]. It should be noted that the CaS (−126.03 kJ/mol) initially formed in the molten steel is easily transformed back into CaO [60,61]. The reaction for the formation of TiN (65.09 kJ/mol) has a positive Gibbs free energy change at 1873 K. Consequently, it cannot be formed spontaneously in molten steel. Figure 3a shows the precipitation curves during the cooling process, calculated using Jmatpro (Version: 7.0) thermodynamic software. It has been demonstrated that nitrides begin to precipitate and grow around 1400 °C and sulfides begin to precipitate and grow around 1300 °C; in addition, a small number of oxides would precipitate. Carbonitrides precipitate at a low temperature, about 1100 °C, and it is difficult to coarsen them into micron particles. CaS and TiN are primarily precipitated on the surface of existing inclusions at a later stage of the solidification process. The different morphologies of CaS observed in the present immersion experiments depend on the diversity of prior oxide inclusions [60].

During the heating process of welding, small precipitations such as TiN and NbC redissolve into the matrix, which reprecipitates again during the cooling process [37,43,63]. This is well described by the expression of solid solubility product at the specific precipitations, as shown in Equation (13):

(13) lg{[X]·[Y]}=A−B/T

where *X* and *Y* denote the equilibrium solubility of a substance in the steels at the required temperature, (wt %), respectively. *A* and *B* are constants and *T* is the absolute temperature, (K). In this paper, the potential precipitations may include NbC, NbN, TiN, TiC, MnS, etc. Table 3 displays the equilibrium relationship between the solid solubility product and temperature for these precipitations. Figure 3b depicts the precipitation curve of these precipitations. According to the Ostwald aging mechanism, tiny nanoprecipitations tend to redissolve in the matrix in the welding thermal cycle, owing to the high heating temperature (peak temperature of 1320 °C), and reprecipitate during the cooling process. Meanwhile, at this peak temperature, a limited number of precipitations grow larger [64]. According to EDS analysis, the precipitated nanoparticles are mostly irregular TiN.

During the welding thermal cycle, nanoparticles exhibit solid–solution behavior whereas inclusions, particularly oxide inclusions, have high melting points and are almost unaffected by the welding thermal cycle [37,42], which distinguishes the CGHAZ from the base metal. It should be emphasized that the role of nanoprecipitations plays a major part in precipitation strengthening and fine grain strengthening in steels; these are easily submerged by corrosion products when pitting occurs [64].

**Figure 4:**
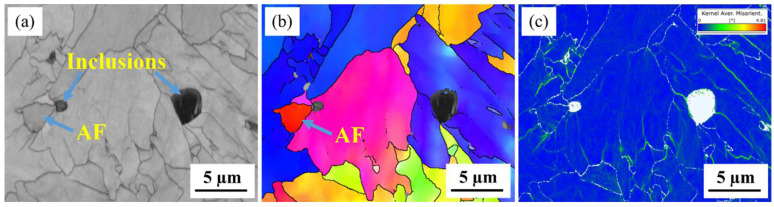
EBSD characterization of the same area containing complex inclusions. (**a**) IQ map, (**b**) IPF map and (**c**) KAM map.

The degree of the local stress concentration or lattice distortion was characterized using kernel average misorientation (KAM) [29,65]. Figure 4 reveals that acicular ferrite grains with varied crystal orientations formed around the inclusions, while the KAM map revealed that a relatively smaller strain concentration exists around the composite inclusion, with or without formed acicular ferrite, as compared to the grain boundaries.

### 3.2. Local Corrosion Induced by the Composite Inclusions in CGHAZ

#### 3.2.1. Potentiodynamic Polarization Tests

**Figure 5:**
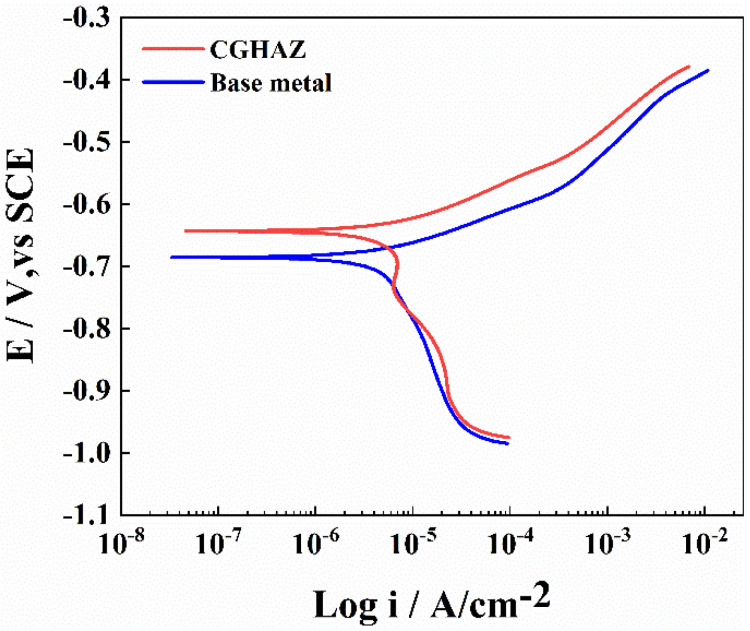
Potentiodynamic polarization curves of the base metal and CGHAZ (100 kJ/cm) of the tested steels in 3.5 wt.% NaCl solution.

**Figure 6:**
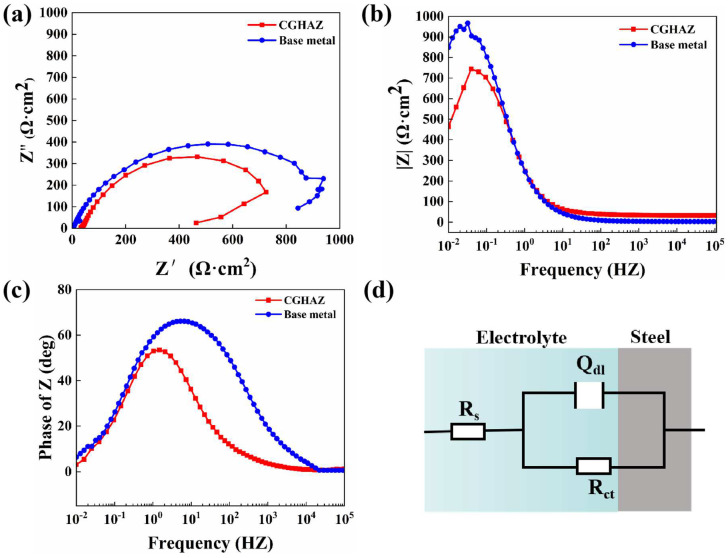
The impedance diagrams for the base metal and CGHAZ in 3.5 wt.% NaCl. (**a**) Nyquist plots, (**b**,**c**) Bode plots and (**d**) proposed equivalent circuit.

**Figure 7:**
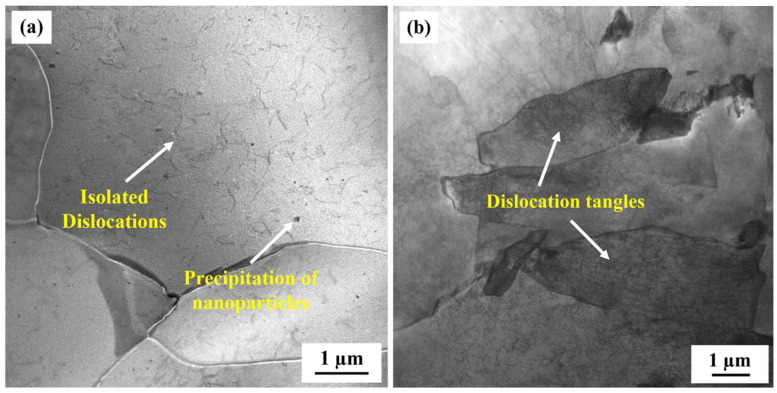
TEM morphologies of the tested steel; (**a**) base metal and (**b**) CGHAZ.

To compare the corrosion rates of the base metal and the CGHAZ of the tested steel, a potentiodynamic polarization test was conducted in 3.5 wt.% NaCl solution (Figure 5). The anode curves of CGHAZ and BM have almost the same shape; this may be related to the fact that there is no passive film on the surface of carbon steel when corrosion occurs. The anodic dissolution here corresponds to iron dissolution. There are two current peaks in the cathode polarization of CGHAZ, which may affect the surface state of the sample due to the larger amount of defects formed during the welding thermal cycle [38,66], the higher stress concentration caused by phase transformation and the complexity of the microstructure formed during the cooling stage [40], thereby affecting the oxygen adsorption process in the cathodic reaction. Table 4 displays the related values of corrosion current (i_corr_) and corrosion potential (E_corr_). The corrosion current density of the CGHAZ is one order of magnitude larger than that of the base metal. Electrochemical impedance spectroscopy (EIS) of BM and of the CGHAZ was performed to better understand the corrosion phenomenon (Figure 6). The Nyquist diagram shows that the curves of BM and the CGHAZ both presented a typical semicircle, indicating that there was only a double layer capacitance between the sample and the solution. As a result, an *R*_s_ (*Q*_dl_*R*_ct_) equivalent circuit diagram was chosen. *R*_s_ represents the solution resistance, *R*_ct_ represents the charge transfer resistance, and *Q*_dl_ represents the double capacitance. The diameter of the arc in the Nyquist diagram reflects the charge transfer resistance. In general, the larger the diameter of the arc, the better the corrosion resistance of the steel. Table 5 summarizes the results obtained from the EIS plots. It can be seen that the *R*_ct_ value of BM is greater than that of the CGHAZ, indicating that BM has better corrosion resistance. In this paper, the microstructure of the base metal is a combination of ferrite and pearlite, while the microstructure of the CGHAZ is a mixture of acicular ferrite and granular bainite. As illustrated in Figure 7, for instance, the base matrix has a lower dislocation density, whereas theCGHAZ exhibits a higher dislocation density.

#### 3.2.2. Immersion Test

**Figure 8:**
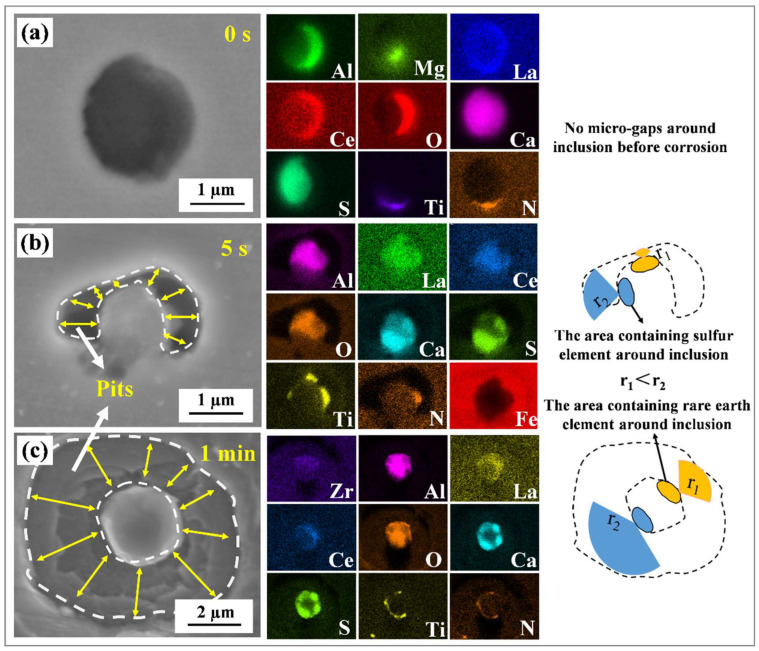
SEM images and EDS results of the immersion test. (**a**) Immersion for 0 s, (**b**) immersion for 5 s and (**c**) immersion for 1 min.

Figure 8a displays the typical morphology—before the immersion test, without any microgaps around the inclusions—of one of the inclusions of interest. As indicated in Figure 8b, a slight dissolution of the matrix occurred around the inclusions after a 5 s immersion. Meanwhile, an anisotropy was observed in the matrix dissolution rate around the inclusions. In this study, the sulfide was identified as CaS. Using SEM-EDS analysis in conjunction with the immersion test, it was shown that the matrix near the CaS at the inclusion surface exhibited a relatively larger corrosion rate than that near the rare earth complex oxides (such as LaAlO_3_) at the initial stage of the pitting corrosion, i.e., r1 < r2. The matrix around the inclusions further dissolved with the ongoing immersion test (Figure 8b,c) and formed ring-shaped pits due to the weakened anisotropy. Therefore, since the sulfides in the inclusions are susceptible to hydrolysis and produce acidic solutions, which accelerates the pitting occurrence, the sulfide attached to the oxide surface is easily transformed into the starting point of the pitting corrosion [9,14].

**Figure 9:**
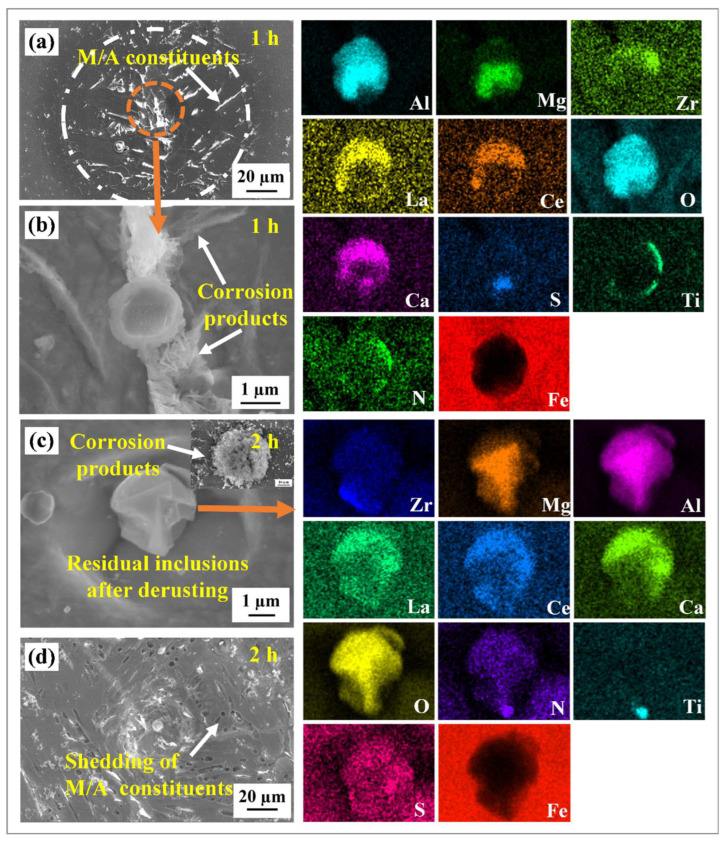
SEM images and EDS results of the immersion test; (**a**,**b**) immersion for 1h and (**c**,**d**) immersion for 2 h.

Figure 9 displays the corrosion morphology of the composite inclusions/matrix after 1 h and 2 h of the immersion test. After the immersion test for 1 h, obvious local corrosion phenomenon occurred around the inclusion, forming a local characteristic region composed of a residual inclusion, dissolved cavity and near-circular corrosion area (Figure 9a). The enlarged area of inclusion revealed minor amounts of flocculent deposits around the inclusion, which indicated that serious localized corrosion occurred around the inclusion (Figure 9b). Combined with SEM-EDS analysis, only a small amount of sulfur element was detected on the inclusions; the residual elements may form inclusions such as (Mg–Al)–Ox, (RE–Zr–Al–Ca)–Ox and TiN. The corrosion products on the sample surface increased significantly after 2 h of immersion testing. According to the SEM results, not all the inclusions were completely covered by the corrosion products. This is attributed to the varying particle sizes of the inclusions. Figure 9c displays the representative morphology of the inclusions after de-rusting. The irregular shape of the residual inclusions and the steeper holes around the inclusions may be related to the faster expansion rate of Cl^-^ in a vertical direction. Furthermore, the morphology of the pits exhibits behavior ranging from near-circular to anisotropic (after rust removal). EDS did not detect any sulfur elements, indicating that all sulfides were completely dissolved, while compounds such as (Mg–Al)–Ox, (RE–Zr–Al–Ca)–Ox and TiN remained undissolved.

Furthermore, after immersion for 1 h, a small amount of corrosion products were found on the surface of the M/A island distributed around the inclusion in the near-circular corrosion area, implying that the corrosion attack occurred in this region (Figure 9a). The micropores were observed around the inclusion after 2 h of immersion (Figure 9d). The microcouples were found to be formed between the M/A constituents and the ferrite matrix, with the ferrite matrix acting as the anode phase and the M/A constituents as the cathode phase [15,46]. The M/A island gradually fell off with the corrosion dissolution of the ferrite matrix.

#### 3.2.3. Elastic Mechanical Properties Calculated by First-Principles Modeling

The elastic mechanical properties of the inclusions involved in this paper are shown in Table 6 and were determined using the first-principles calculation method. According to calculation results, LaAlO_3_ and La_2_O_7_Zr_2_ have lower bulk modulus than Al_2_O_3_ (249.54) and ZrO_2_ (271.06), and the bulk modulus of LaAlO_3_ (192.61) is closer to that of the iron matrix (194.76). The results indicate that LaAlO_3_ has excellent mechanical compatibility with the matrix. Numerous studies have demonstrated that the visible microcrevices and high-density dislocations between Al_2_O_3_ and the matrix are due to the significantly higher hardness and irregular shape of Al_2_O_3_ compared with the iron matrix [29,67]. Inclusions, such as CaO (114.11), MgO (165.84), TiN (175.02) and CaS (57.05), exhibit lower bulk modulus than the matrix. Rare earth elements can form rare earth oxides and composite inclusions, such as LaAlO_3_ and La_2_O_7_Zr_2_, due to their stronger affinity for oxygen than Zr, Ti and other deoxidizers [31]. The spheroidization and softening effects of rare earth elements on the inclusions can reduce the stress concentration around the inclusions [32,67,68]. This is consistent with the absence of microcracking that was observed between the inclusions and the matrix (Figure 4 and Figure 8a).

#### 3.2.4. Inclusion Conductivity Property Calculated by First-Principles Modeling

**Figure 10:**
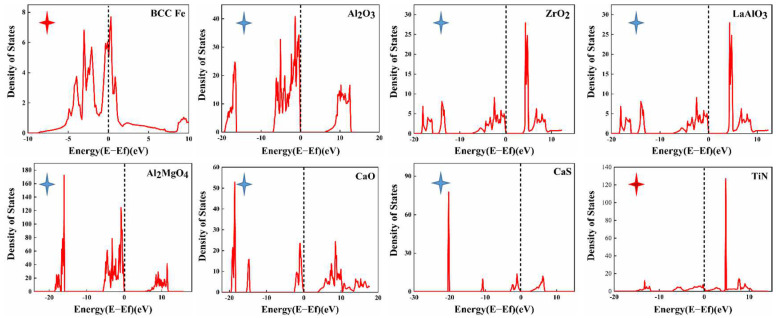
Density of states (DOS) analysis of the inclusions (the black dotted line represents the Fermi energy level).

In the present work, density of states of the inclusions of interest were calculated to determine the electrical conductivity. It can be seen that the Fe matrix and TiN (red star) have metallic nature, whereas Al_2_O_3_, ZrO_2_, LaAlO_3_, La_2_O_7_Zr_2_, MgO, CaO, CaS and Al_2_MgO_4_ (blue star) exhibit the properties of insulators (Figure 10). The matrix and TiN exhibit electrical conductivity, whereas the other oxidation or sulfide inclusions exhibit the properties of insulators or semiconductors. TiN holds intact in the immersion test, which could be attributed to its insolubility in strong acids at room temperature and pressure [69,70]. In summary, the composite inclusions in the test steel will not form a microcouple effect with the matrix.

#### 3.2.5. Work Function Calculated by First-Principles Modeling

**Figure 11:**
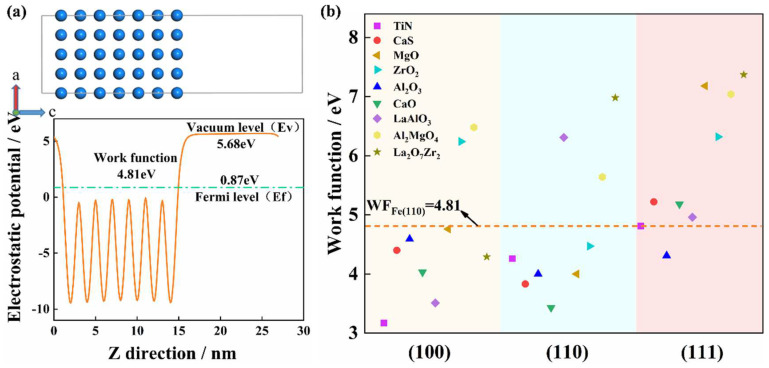
Calculated work function of the inclusions and Fe matrix. (**a**) Electrostatic potential curve of Fe (110) slab model, (**b**) work function of the different crystal planes (low index) of the inclusions.

Figure 11 presents the work functions of inclusions and the matrix involved in this paper. The work function is defined as the energy difference between the Fermi level and the vacuum level (Figure 11a) of a slab model and represents the minimum energy required to remove an electron from the sample surface [32,67]. Since Fe (110) surface has the lowest surface energy with the largest possible exposed face, the work function of the Fe (110) surface is utilized as the baseline in this paper [71]. The work functions of the low-index crystal surfaces of Al_2_O_3_, CaO, MgO, TiN and CaS are almost relatively lower, while the work functions of the low-index crystal surfaces of LaAlO_3_, La_2_O_7_Zr_2_, ZrO_2_ and Al_2_MgO_4_ are significantly higher than the work function of the Fe (110) surface (Figure 11b). The work function of the typical inclusion combined with the first-principles calculation results shows that the composite inclusion containing rare earth elements has a higher work function, which implies that it is less likely to act as an anode phase compared to CaS. Furthermore, the work function value corresponds to the phenomena observed in the immersion test, in which CaS was preferentially dissolved and (Mg–Al)–Ox and (RE–Zr–Al–Ca)–Ox remained undissolved (Figure 9d). The addition of rare earth elements can improve inclusion corrosion resistance [18].

### 3.3. Mechanism of Local Corrosion Induced by Zr–Ti–Al–RE Composite Inclusions in CGHAZ

**Figure 12:**
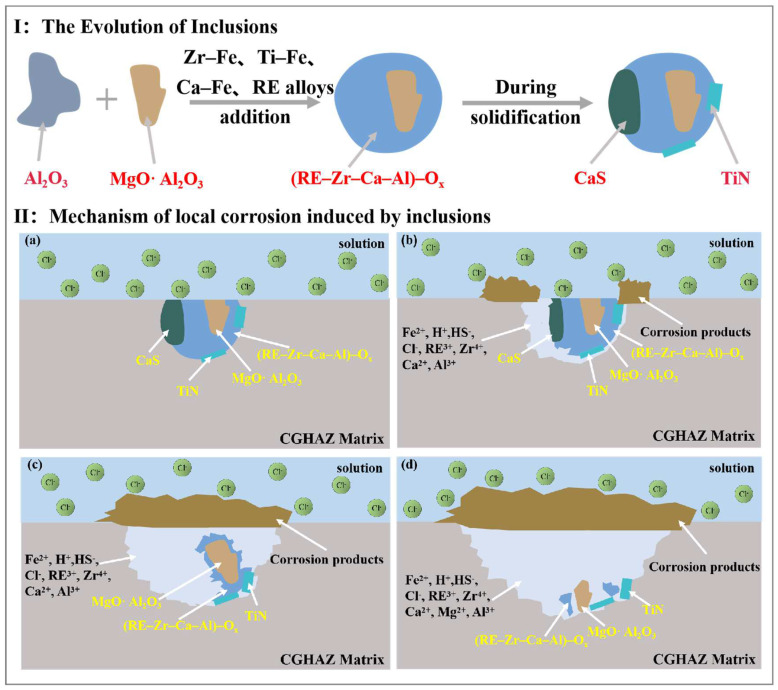
(**I**) Schematic diagram of the inclusion evolution process and (**II**) pitting initiation and propagation process induced by the composite inclusions. (**a**) before immersion, (**b**) pitting initiation, (**c**,**d**) pitting propagation.

Figure 12I illustrates the evolution process of the inclusions involved in this paper. The addition of Zr–Fe, Ti–Fe, Ca–Fe, and RE alloys during the smelting process modified the Al_2_O_3_ inclusions into composite inclusions. CaS and TiN precipitated on the previously formed inclusions at a later stage of solidification, ultimately forming composite inclusions with a core–shell structure [12,14,72]. Figure 12II illustrates the schematic diagram of the initiation and propagation of the pitting corrosion triggered by these composite inclusions in the tested steel. As mentioned above, the mechanisms of galvanic coupling corrosion and the crevice and stress corrosion formed between the inclusions and the matrix could be excluded in this study. Since the composite inclusions are exposed to a corrosive environment with Cl^-^ (Figure 12IIa), CaS with poor stability will preferentially hydrolyze to produce H^+^ and HS^-^ by anodic reactions (Equations (14) and (15)). As the reaction progresses, the matrix begins to undergo a hydrolysis reaction (Equations (16) and (17)), resulting in further acidification in the pits and further dissolution of CaS [9,14]. The composite inclusion (RE–Zr–Al–Ca)–Ox and the surrounding matrix were gradually dissolved (Equation (18) under the attack of the increasing H^+^ with the continued dissolution of CaS. Meanwhile, corrosion products produced by cathodic reactions (Equations (19)–(21)) gradually accumulated at the edge of pits (Figure 12 IIb). CaS was completely dissolved, forming and propagating stable pits with the total corrosion products covering the upper layer. To maintain electrical neutrality, a large amount of Cl^-^ will enter the pitting hole, resulting in the formation of a self-catalytic acidification-occluded corrosion cell (Figure 12IIc). Furthermore, some generated cations, such as Ca^2+^, Zr^4+^, Al^3+^ and RE^3+^, hydrolyze further, resulting in a drop in the pH value [13,30,32], causing a strong acidic environment in the pits and accelerating the dissolution of the composite inclusions ((RE–Zr–Al–Ca)–Ox and Al_2_MgO_4_) (Figure 12IId). TiN inclusion is stable, which is primarily related to its insolubility in strong acids [69]. With the dissolution of (RE–Zr–Al–Ca)–Ox, Al_2_MgO_4_ and TiN inclusions would fall to the bottom of the pits. The anodic and cathodic reactions involved during the pitting initiation and propagation of the Zr–Ti–Al–RE composite inclusions in the tested steel can be summarized as follows:

Anodic reactions:

(14) $${\mathrm{CaS}+2H}_{2}O\to {\mathrm{Ca}}^{2+}{+2\mathrm{OH}}^{-}{+H}_{2}S$$

(15) $$2H_{2}S⇌{2H}^{+}{+2\mathrm{HS}}^{-}$$

(16) $$\mathrm{Fe}-{2e}^{-}\to {\mathrm{Fe}}^{2+}$$

(17) $${\mathrm{Fe}+2H}_{2}O\to \mathrm{Fe}{\left(\mathrm{OH}\right)}_{2}{+2H}^{+}$$

(18) $$\left(\mathrm{RE}–\mathrm{Zr}–\mathrm{Mg}–\mathrm{Al}–\mathrm{Ca}\right)O_{X}{+H}^{+}\to {\mathrm{RE}}^{3+}{+\mathrm{Zr}}^{2+}{+\mathrm{Mg}}^{2+}{+\mathrm{Al}}^{3+}{+\mathrm{Ca}}^{2+}{+H}_{2}O$$

Cathodic reactions:

(19) $$O_{2}{+2H}_{2}{O+e}^{-}\to {4\mathrm{OH}}^{-}$$

(20) $${\mathrm{Fe}}^{2+}+2{\mathrm{OH}}^{-}\to \mathrm{Fe}{\left(\mathrm{OH}\right)}_{2}$$

(21) $$4\mathrm{Fe}{\left(\mathrm{OH}\right)}_{2}+O_{2}{+2H}_{2}O\to 4\mathrm{Fe}{\left(\mathrm{OH}\right)}_{3}$$

The matrix around the spherical inclusions was dissolved to produce cations in the early stage of the pitting corrosion, and Cl^−^ migrated to the anode and diffused in a horizontal direction under the concentration gradient. This resulted in the nearly circular shape of the pits, which eventually produced corrosion products covering the pits [9,13]. Following the formation of the self-catalytic acidification-occluded corrosion cell, the acidic environment accelerates the dissolution of the composite inclusions. However, the findings of the work function analyses of the inclusions in this work demonstrate that the inclusions display different corrosion resistances and the cations exhibit different chemical hydrolysis abilities. These two factors lead to asynchrony of the hydrolysis reaction, resulting in anisotropy of the pitting area around the inclusions.

In conclusion, compared with Al_2_O_3_ inclusions introduced by the traditional Al deoxidization technology, the addition of rare earth can effectively modify the characteristics of the inclusions.

## 4. Conclusions

1. The inclusions and microstructures in the Zr–Ti–Al–RE deoxidized HSLA steels were modified. The formed spherical Zr–Ti–Al–RE composite inclusions have a core–shell structure with (Zr–Mg–Al–Ca–RE)–Ox at the center and CaS and TiN at the periphery.
2. Acicular ferrite was formed at specific inclusions in the CGHAZ, which could improve the mechanical properties.
3. The spheroidization and softening effects of rare earth elements on the inclusions can lower the stress concentration around the inclusions and increase the local corrosion resistance of the tested steel. Furthermore, the inclusions containing rare earth elements have a significantly higher work function than the matrix, which further reduces the corrosion tendency of the inclusions to undergo anodic dissolution.
4. The initiation of pitting corrosion from composite inclusions is triggered by the chemical dissolution of CaS within the composite inclusions. Then, (RE–Zr–Al–Ca)–Ox started to dissolve until the composite inclusion Al_2_MgO_4_ region was exposed. Finally, the undissolved Al_2_MgO_4_ and TiN fell to the bottom of the pit. The composite spherical inclusions gradually dissolved and transformed into irregular shapes as the immersion duration increased. The addition of rare earth can effectively reduce the tendency for pitting corrosion in a CGHAZ in a marine environment.

## Figures and Tables

**Table 1:** Chemical composition of the tested steel (wt, %).

C	Si	Mn	Nb	Al + Ca + Zr + Ti + RE	Fe
0.05	0.17	1.55	0.034	0.04	Bal.

**Table 2:** Gibbs free energy of the inclusion formation reaction at 1873 K [57,58,62].

No.	Reactions	*ΔG^θ^* (J/mol)	*ΔG* (kJ/mol)
1	2[Al] + 3[O] = (Al_2_O_3_)	−1,682,927 + 323.240 T	−734.33
2	[Mg]+2[Al] + 4[O] = (MgO∙Al_2_O_3_)	−1,848,696 + 574.144 T	*ΔG^θ^* = −773.32
3	[Zr] + 2[O] = (ZrO_2_)	−845,532 + 266.100 T	−156.09
4	2[Ti] + 3[O] = (Ti_2_O_3_)	−1,072,872 + 346.000 T	−273.72
5	[Ca] + [O] = (CaO)	−138,227 − 63.000 T	−96.36
6	[Ca]+6[Al] + 4[O] = [CaO∙Al_2_O_3_]	−1,023,637 + 142.120 T	−9458.40
7	2[La] + 3[O] = (La_2_O_3_)	−542,531 +124.150 T	−610.85
8	3[Zr] + 2(Al_2_O_3_) = 4[Al] + 3(ZrO_2_)	−8,233,279+ 464.510 T	−7298.12
9	3[Zr] + 2(Ti_2_O_3_) = 4[Ti] + 3(ZrO_2_)	−1,073,389 + 538.830 T	−11.99
10	[La] + 3[O] + [Al] = (LaAlO_3_)	−801,616 + 129.000 T	−646.15
11	[Ce] + (Al_2_O_3_) = (CeAlO_3_)+[Al]	−423,900 − 247.300 T	−833.13
12	[Ca] + [S] = (CaS)	−542,531 + 124.150 T	−126.03
13	[Ti] + [N] = (TiN)	−307,620 + 113.400 T	65.09

**Table 3:** The solubility expressions for the precipitations [63,64].

No.	Precipitation	Solubility Product
1	NbC	lg{[Nb]·[C]} = 2.26−6770/T
2	NbN	lg{[Nb]·[N]} = 2.80−8500/T
3	TiC	lg{[Ti]·[C]} = 2.75−7000/T
4	TiN	lg{[Ti]·[N]} = 0.32−8000/T
5	MnS	lg{[Mn]·[S]} = 5.02−11625/T

**Table 4:** Electrochemical corrosion parameters of the tested steels.

Samples	i_corr_ (A·cm^2^)	E_corr_ (V vs. SCE)
Base metal	9.31 × 10^−6^	−0.69
CGHAZ	1.02 × 10^−5^	−0.64

**Table 5:** Electrochemical parameters de1duced by EIS method in 3.5 wt.% NaCl solution.

Samples	*R*_s_ (Ω·cm^−2^)	Y_0_ (S·secn·cm^−2^)	n	*R*_ct_ (Ω·cm^−2^)
CGHAZ	33.78	0.0008772	0.78	763.2
Base metal	20.31	0.0007096	0.87	948.3

**Table 6:** Calculated mechanical properties of the inclusions and Fe matrix.

Inclusions	Bulk Modulus (GPa)	Shear Modulus (GPa)	Young’s Modulus (GPa)	Poisson’s Ratio
Fe (BCC)	194.76	81.52	214.61	0.32
Al_2_O_3_	249.54	152.76	380.61	0.25
ZrO_2_	271.06	109.14	288.68	0.32
La_2_O_7_Zr_2_	165.83	60.56	161.95	0.34
LaAlO_3_	192.61	121.10	300.35	0.24
CaO	114.11	78.81	192.19	0.22
MgO	165.83	127.64	304.73	0.19
CaS	57.05	39.07	95.42	0.22
TiN	175.02	79.89	208.01	0.30

## Data Availability

The raw processed data required to reproduce these findings cannot be shared at this time as the data also form part of an ongoing study.
